# Supplementary material for: A negative feedback loop between TET2 and leptin in adipocyte regulates body weight
Source: Nat Commun. 2024 Apr 1;15:2825. doi: 10.1038/s41467-024-46783-x (PMC10985112; doi:10.1038/s41467-024-46783-x)
Supplement: Supplementary file 1 — Supplementary Information [file 41467_2024_46783_MOESM1_ESM.pdf]

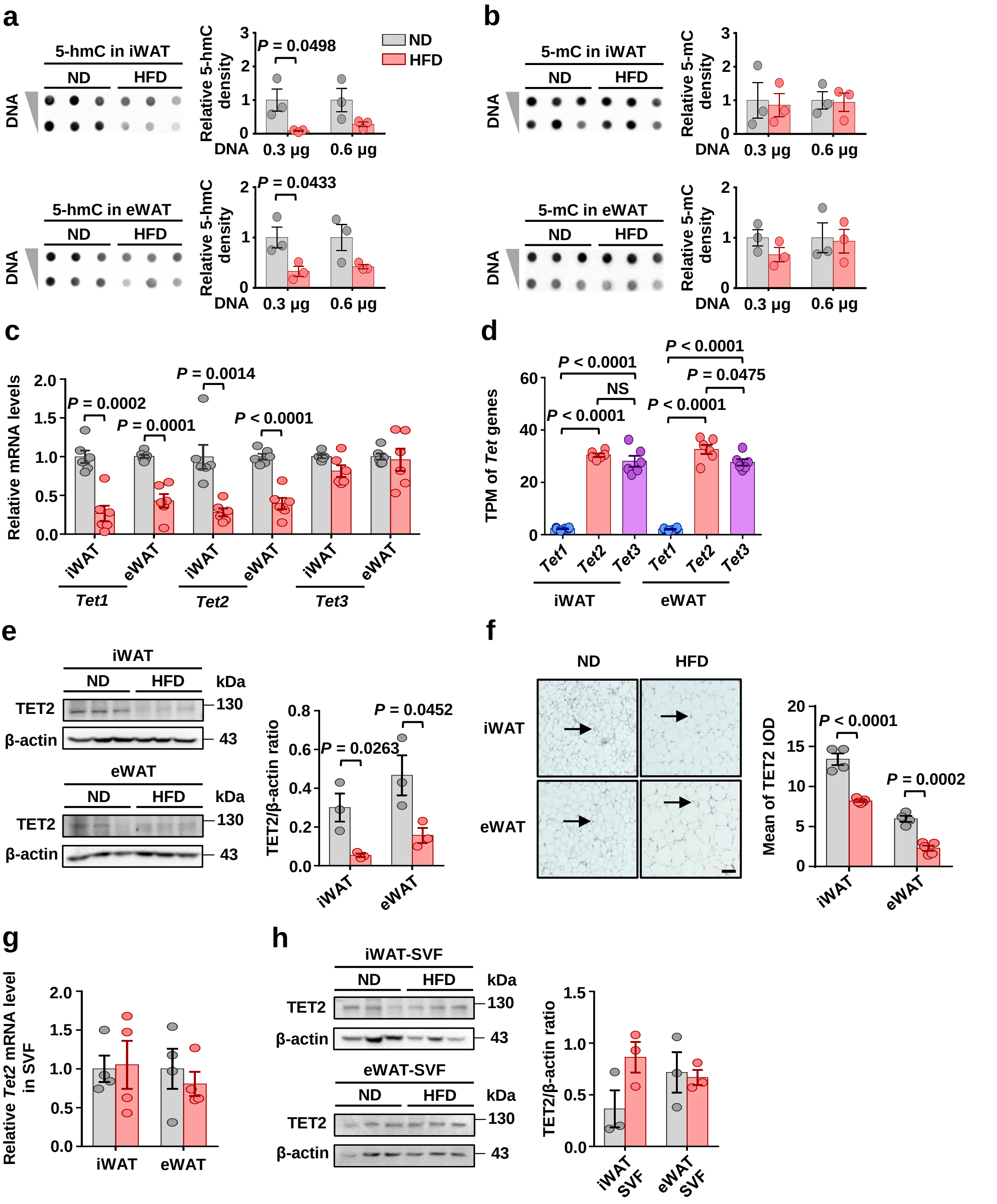

Figure S1. Obesity decreases DNA hydroxymethylation and TET2 levels in white adipose tissues

(a-b) Genomic 5-hmC (a) and 5-mC (b) levels in iWAT and eWAT from the C57BL/6J male mice fed either ND or HFD for 12 weeks (n = 3 mice/group).

(c) mRNA levels of *Tet1*, *Tet2*, and *Tet3* relative to *β-actin* in iWAT and eWAT from the mice shown in a (n = 6 mice/group).

(d) Relative mRNA expression of *Tet1*, *Tet2*, and *Tet3* in iWAT and eWAT from the C57BL/6J male mice fed with ND (Published bulk RNA-seq data: GSE132706; n = 6 mice/group).

(e) Representative immunoblot images of TET2 in iWAT and eWAT from the mice shown in a and densitometry analysis. β-actin was used as a loading control (n = 3 mice/group).
(f) Representative immunohistochemistry images of TET2 in iWAT and eWAT from the mice shown in a (the arrows represent TET2 positive expression, scale bars, 100 μm) and integral optical density (IOD) analysis. (n = 4 ND; n = 5 HFD).
(g) *Tet2* mRNA levels relative to *β-actin* in SVF of iWAT and eWAT from the mice shown in a (n = 4 ND/HFD, 1 ND sample was obtained by pooling samples from two mice).
(h) Representative immunoblot images of TET2 in SVF of iWAT and eWAT from the mice shown in a and densitometry analysis. β-actin was used as a loading control (n = 3 mice/group). All data are presented as mean ± SEM. P values are indicated on the graph. Statistical values are determined by two-sided unpaired Student's t-test. Source data are provided as a Source Data File.

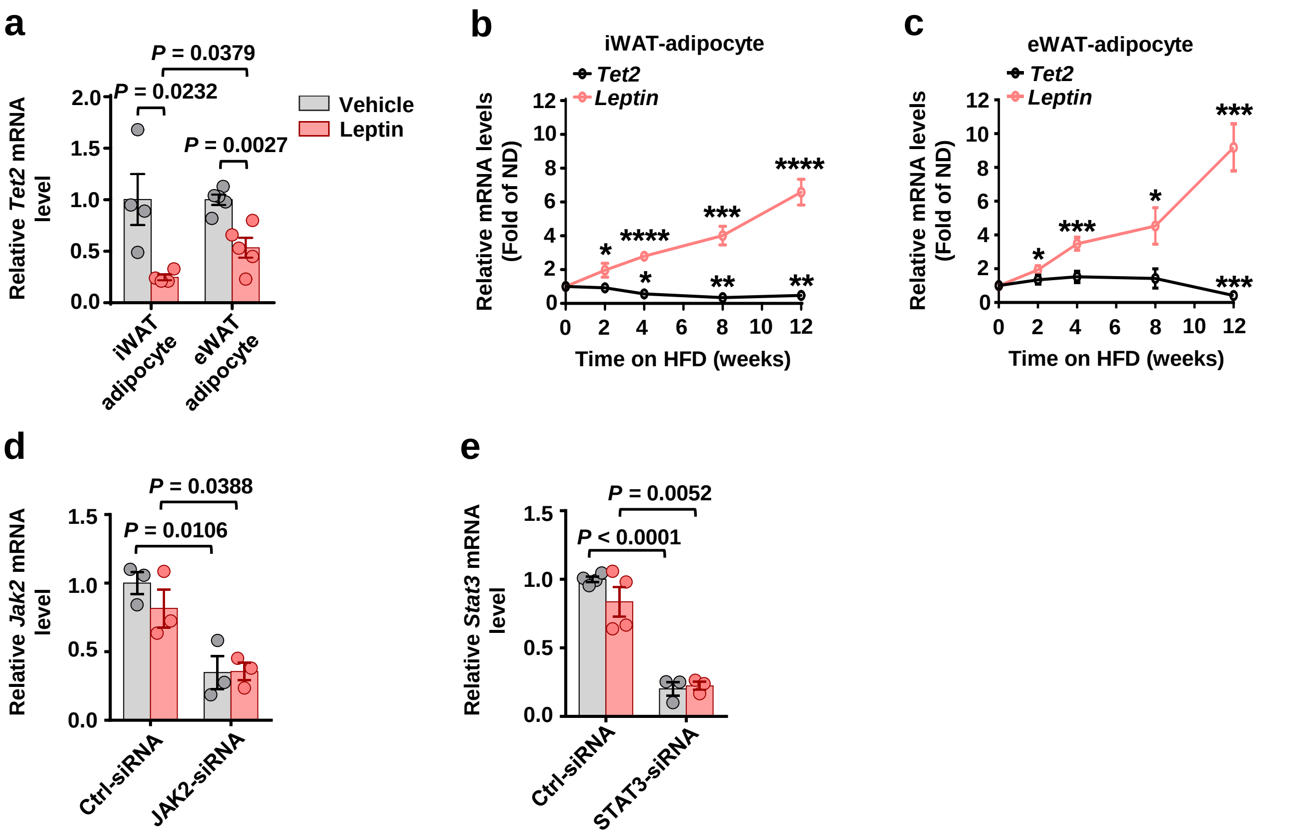

Figure S2. Leptin inhibits adipocyte Tet2 expression in primary adipocytes and differentiated adipocytes

(a) *Tet2* expression in mature adipocytes from iWAT and eWAT treated with vehicle or leptin for 24 h (iWAT adipocyte: n = 4 mice/group; eWAT adipocyte: n = 5 mice/group).

(b) *leptin* and *Tet2* mRNA levels relative to *36b4* in adipocytes of iWAT from the C57BL/6J male mice fed HFD for 0, 2, 4, 8, and 12 weeks (*Leptin* gene: HFD 0 week: n = 6; HFD 2 weeks: n = 3, $^{*}P = 0.0212$; HFD 4 weeks: n = 6, $^{****}P < 0.0001$; HFD 8 weeks: n = 6, $^{***}P = 0.0003$; HFD 12 weeks: n = 6, $^{****}P < 0.0001$; *Tet2* gene: HFD 0 week: n = 6; HFD 2 weeks: n = 3; HFD 4 weeks: n = 6, $^{*}P = 0.0190$; HFD 8 weeks: n = 6, $^{**}P = 0.0064$; HFD 12 weeks: n = 6, $^{**}P = 0.0041$).

(c) *leptin* and *Tet2* mRNA levels relative to *36b4* in adipocytes of eWAT from the C57BL/6J male mice fed HFD for 0, 2, 4, 8, and 12 weeks (*Leptin* gene: HFD 0 week: n = 5; HFD 2 weeks: n = 4, $^{*}P = 0.0156$; HFD 4 weeks: n = 5, $^{***}P = 0.0004$; HFD 8 weeks: n = 5, $^{*}P = 0.0119$; HFD 12 weeks: n = 5, $^{***}P = 0.0004$; *Tet2* gene: HFD 0 week: n = 4; HFD 2 weeks: n = 4; HFD 4 weeks: n = 4; HFD 8 weeks: n = 4; HFD 12 weeks: n = 5, $^{***}P = 0.0004$).

(d) *Jak2* expression in differentiated 3T3-L1 adipocytes treated with Ctrl-siRNA or JAK2-siRNA for 24 h (n = 3).

(e) *Stat3* expression in differentiated 3T3-L1 adipocytes treated with Ctrl-siRNA or STAT3-siRNA for 24 h (n = 4 Ctrl-siRNA; n = 3 STAT3-siRNA). All data are presented as mean ± SEM. *P* values are indicated on the graph. Statistical values are determined by two-sided unpaired Student's t-test. $^{*}P < 0.05$, $^{**}P < 0.01$, $^{***}P < 0.001$, $^{****}P < 0.0001$ versus HFD 0 week. Source data are provided as a Source Data File.

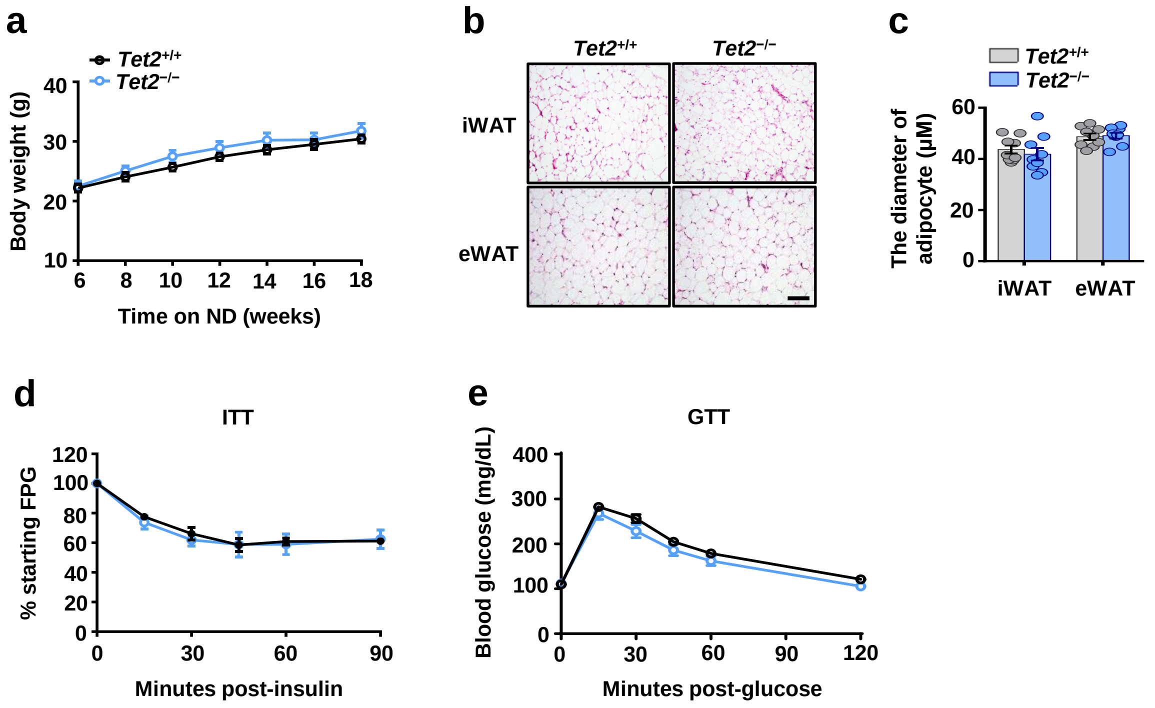

Figure S3: Tet2 deficiency has no effect on insulin sensitivity and glucose tolerance under ND-fed condition

(a) Body weight progression in *Tet2*$^{+/+}$ and *Tet2*$^{-/-}$ mice fed ND for 18 weeks (n = 9 mice/group).

(b) Representative H&E staining of iWAT and eWAT of *Tet2*$^{+/+}$ and *Tet2*$^{-/-}$ mice fed ND for 18 weeks. Scale bars: 50 μm.

(c) The average diameters of adipocytes in *Tet2*$^{+/+}$ and *Tet2*$^{-/-}$ mice fed ND for 18 weeks were analyzed and quantified by the Image-Pro Plus software (n = 9 mice/group).

(d) Insulin tolerance test (ITT) at week 18 of life (n = 9 *Tet2*$^{+/+}$; n = 8 *Tet2*$^{-/-}$).

(e) Glucose tolerance test (GTT) at week 18 of life (n = 9 *Tet2*$^{+/+}$; n = 8 *Tet2*$^{-/-}$). All data are presented as mean ± SEM. Statistical values are determined by two-sided unpaired Student's t-test. Source data are provided as a Source Data File.

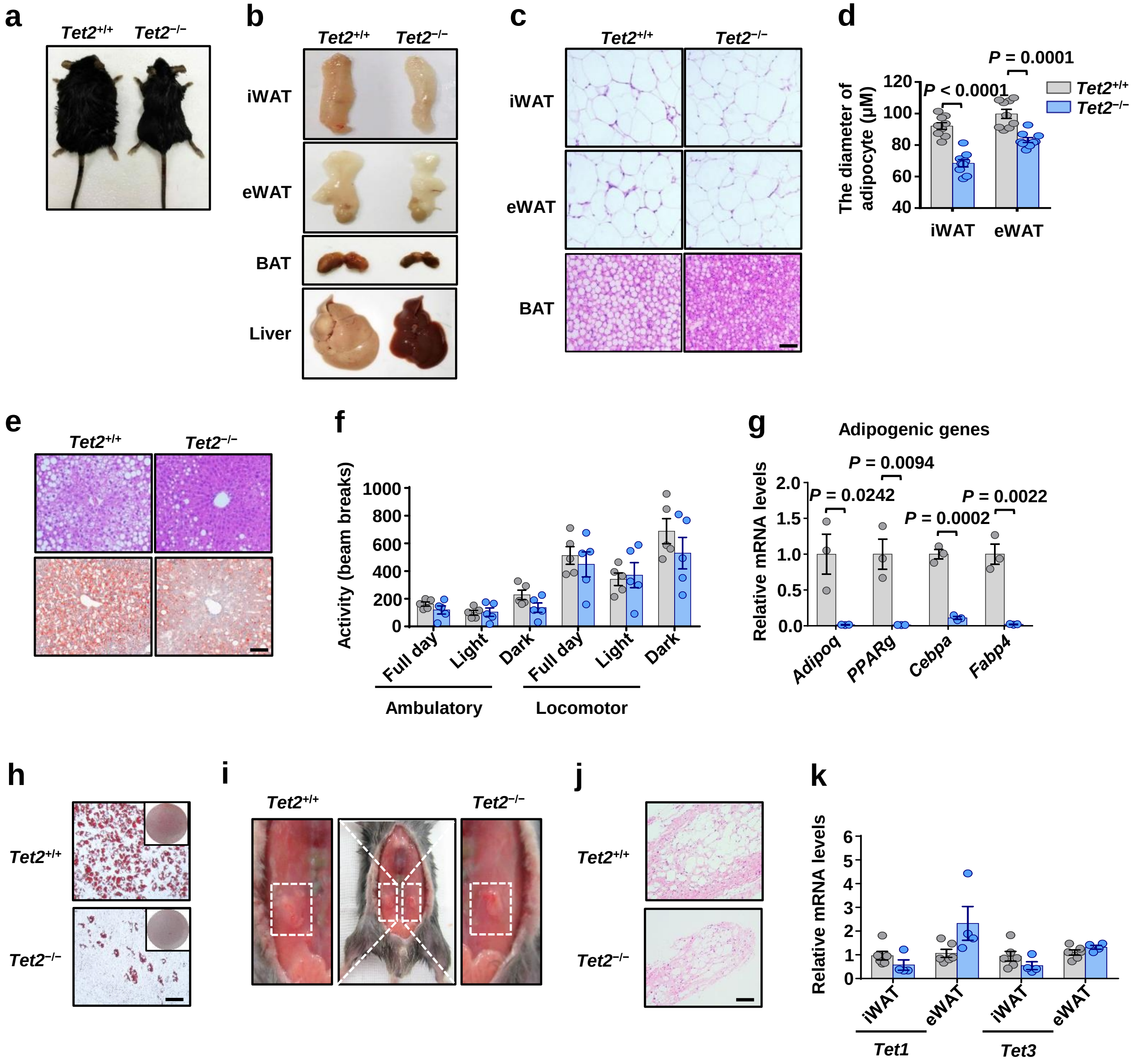

Figure S4: Tet2 deficiency attenuates HFD-induced fat expansion

(a) Representative images of *Tet2*$^{+/+}$ and *Tet2*$^{-/-}$ mice fed HFD for 12 weeks, starting at 6 weeks of age.

(b) Representative images of fat pads and liver collected from *Tet2*$^{+/+}$ and *Tet2*$^{-/-}$ mice fed HFD for 12 weeks.

(c) Representative H&E staining of iWAT, eWAT and BAT of *Tet2*$^{+/+}$ and *Tet2*$^{-/-}$ mice fed HFD for 12 weeks. Scale bars: 50 μm.

(d) The average diameters of adipocytes in *Tet2*$^{+/+}$ and *Tet2*$^{-/-}$ mice fed HFD for 12 weeks were analyzed and quantified by the Image-Pro Plus software (n = 9 mice/group).

(e) Representative H&E and Oil Red O staining of liver of *Tet2*$^{+/+}$ and *Tet2*$^{-/-}$ mice fed HFD for 12 weeks. Scale bars: 50 μm.

(f) Physical activity in *Tet2*$^{+/+}$ and *Tet2*$^{-/-}$ mice after 5 weeks of HFD feeding (n = 5 mice/group).

(g) mRNA levels of adipogenic genes relative to *36b4* in differentiated ASCs from iWAT of *Tet2*$^{+/+}$ and *Tet2*$^{-/-}$ mice (n = 3 mice/group).

(h) Oil Red O staining of differentiated cells in (g). Low (top) and high magnification (bottom) are shown. Scale bar, 50 μm.
(i) Image of implantation sites after 5 weeks of HFD. ASCs from iWAT of *Tet2*$^{+/+}$ and *Tet2*$^{-/-}$ mice were implanted into the abdominal subcutaneous depot in Matrigel.
(j) Microscopy images of Matrigel plug with ASCs from iWAT of *Tet2*$^{+/+}$ and *Tet2*$^{-/-}$ mice in (i). Scale bar, 50 μm.
(k) mRNA levels of *Tet1* and *Tet3* relative to *β-actin* in iWAT and eWAT from *Tet2*$^{+/+}$ and *Tet2*$^{-/-}$ mice fed with ND (n = 6 *Tet2*$^{+/+}$; n = 4 *Tet2*$^{-/-}$). All data are presented as mean ± SEM. Statistical values are determined by two-sided unpaired Student's t-test in (d, g and k), one-way ANCOVA test in (f). Source data are provided as a Source Data File.

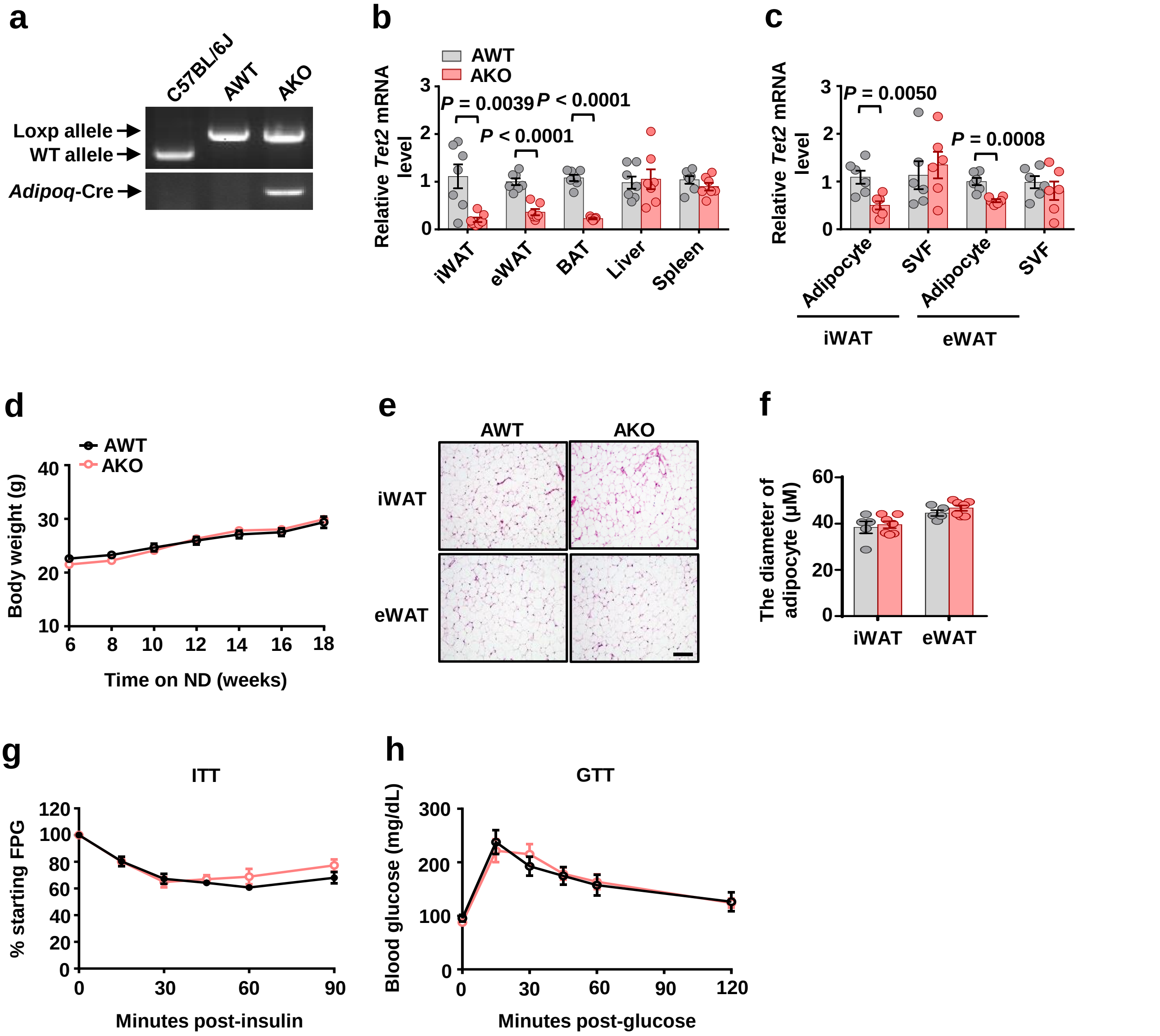

Figure S5: Adipocyte-specific Tet2 deficiency has no effect on insulin sensitivity and glucose tolerance under ND-fed condition

(a) PCR genotyping approach.

(b) *Tet2* mRNA levels relative to *β-actin* in iWAT, eWAT, BAT, liver, and spleen from the AWT and AKO mice fed ND for 18 weeks (n = 7 mice/group).

(c) *Tet2* mRNA levels relative to *36b4* in adipocytes and relative to *β-actin* in SVF of iWAT and eWAT from the mice shown in b (n = 6 mice/group).

(d) Body weight progression in AWT and AKO mice fed ND for 18 weeks (n = 5 AWT; n = 7 AKO).

(e) Representative H&E staining of iWAT and eWAT of AWT and AKO mice fed ND for 18 weeks. Scale bars: 50 µm.

(f) The average diameters of adipocytes in AWT and AKO mice fed ND for 18 weeks were analyzed and quantified by the Image-Pro Plus software (n = 6 mice/group).

(g) ITT at week 18 of life (n = 5 AWT; n = 7 AKO).

(h) GTT at week 18 of life (n = 5 AWT; n = 7 AKO). All data are presented as mean ± SEM. *P* values are indicated on the graph. Statistical values are determined by two-sided unpaired Student's t-test. Source data are provided as a Source Data File.

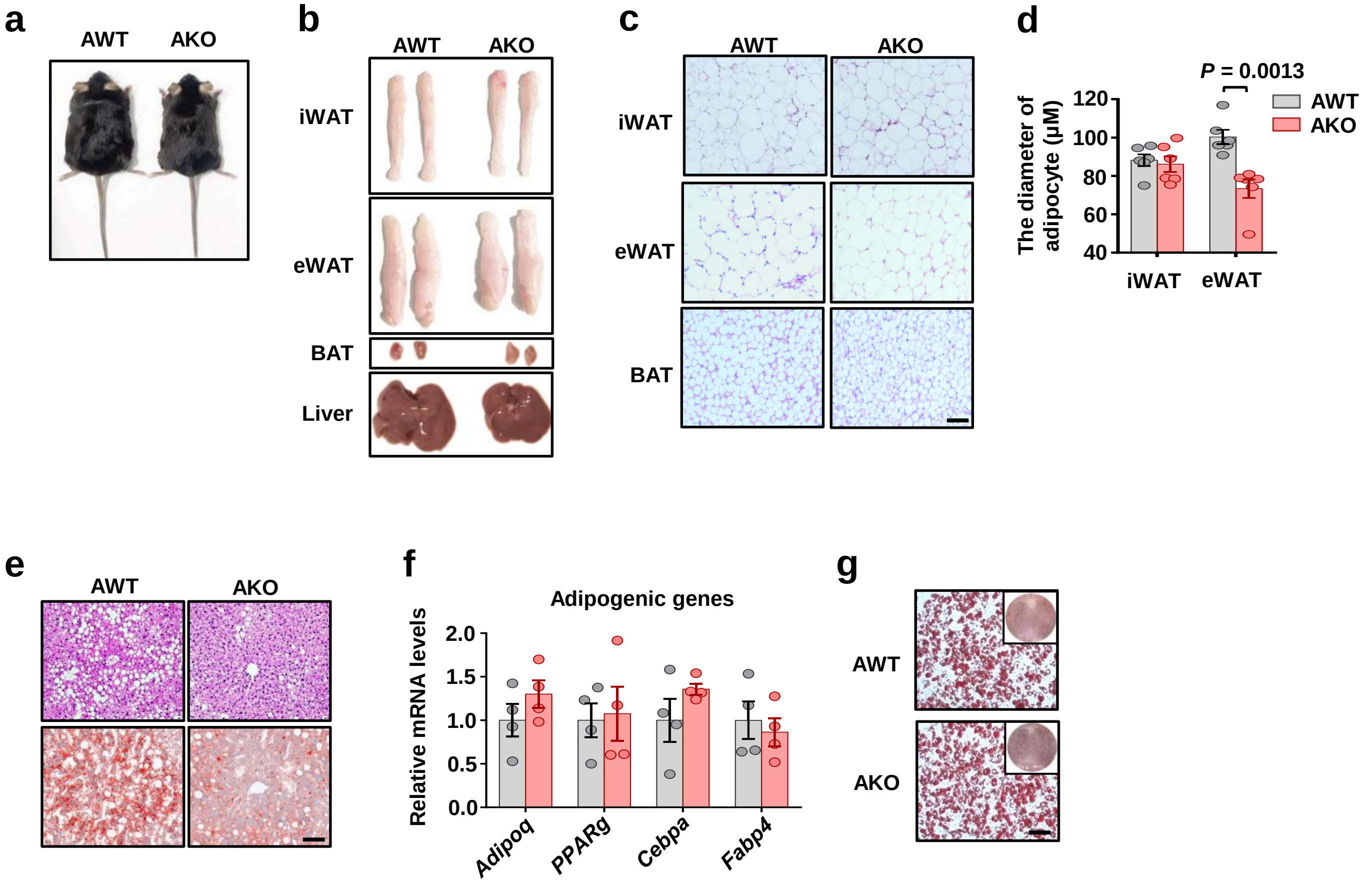

Figure S6: Adipocyte-specific Tet2 deficiency protects against HFD-induced fat expansion

(a) Representative images of AWT and AKO mice fed HFD for 12 weeks, starting at 6 weeks of age.

(b) Representative images of fat pads and liver collected from AWT and AKO mice fed HFD for 12 weeks.

(c) Representative H&E staining of iWAT, eWAT and BAT of AWT and AKO mice fed HFD for 12 weeks. Scale bars: 50 μm.

(d) The average diameters of adipocytes in AWT and AKO mice fed HFD for 12 weeks were analyzed and quantified by the Image-Pro Plus software (n = 6 mice/group).

(e) Representative H&E and Oil Red O staining of liver of AWT and AKO mice fed HFD for 12 weeks. Scale bars: 50 μm.

(f) mRNA levels of adipogenic genes relative to *36b4* in differentiated ASCs from iWAT of AWT and AKO mice (n = 4 mice/group).

(g) Oil Red O staining of differentiated cells in (f). Low (top) and high magnification (bottom) are shown. Scale bar, 50 μm. All data are presented as mean ± SEM. *P* values are indicated on the graph. Statistical values are determined by two-sided unpaired Student's t-test. Source Data are provided as a Source Data File.

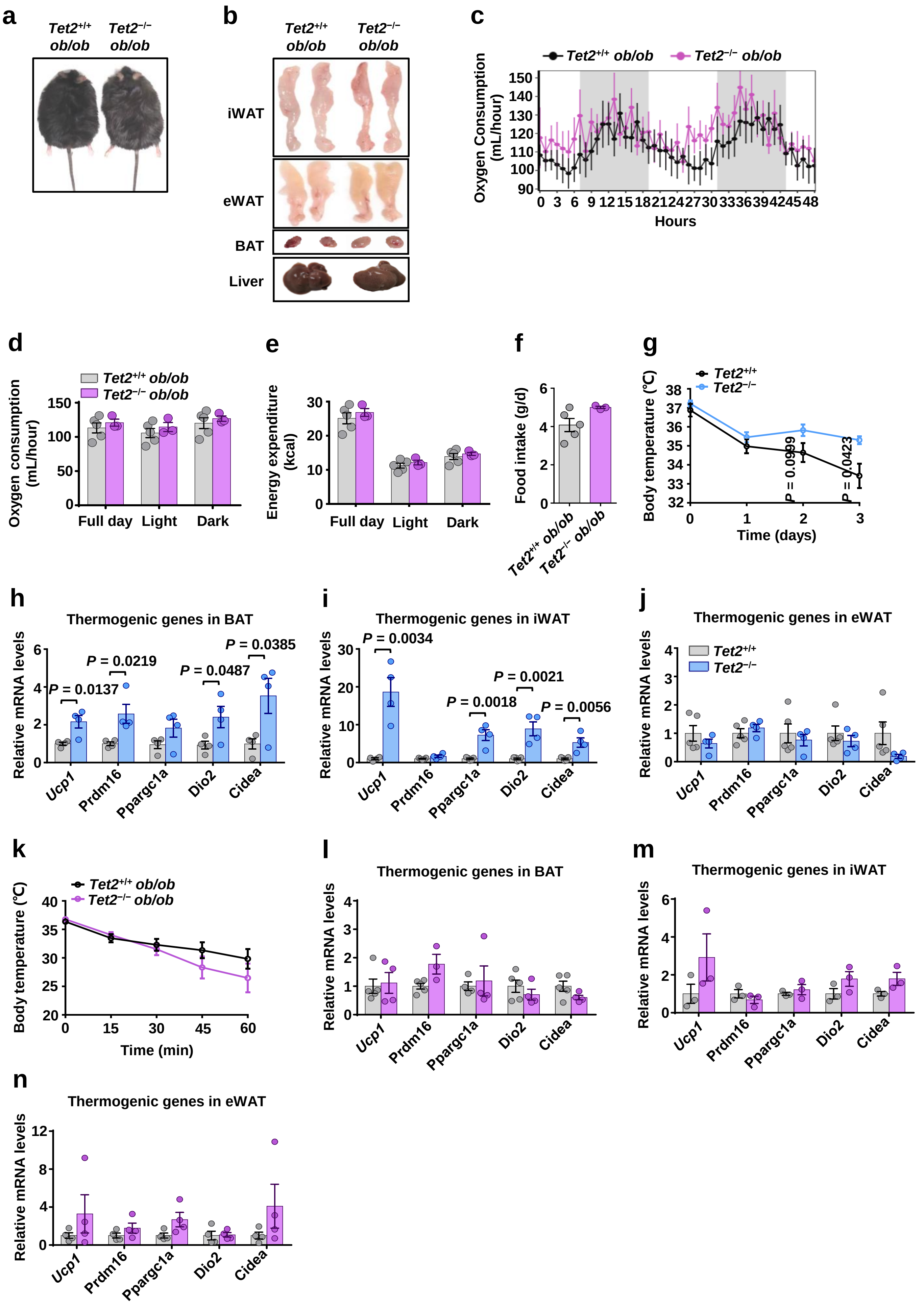
a
Tet2+/+ ob/ob
Tet2−/− ob/ob
b
iWAT
eWAT
BAT
Liver
c
Oxygen Consumption (mL/hour)
Hours
d
Oxygen consumption (mL/hour)
Full day
Light
Dark
e
Energy expenditure (kcal)
f
Food intake (g/d)
g
Body temperature (°C)
Time (days)
P = 0.0999
P = 0.0423
h
Thermogenic genes in BAT
Relative mRNA levels
P = 0.0137
P = 0.0219
P = 0.0487
P = 0.0385
Ucp1
Prdm16
Ppargc1a
Dio2
Cidea
i
Thermogenic genes in iWAT
P = 0.0034
P = 0.0018
P = 0.0021
P = 0.0056
j
Thermogenic genes in eWAT
Tet2+/+
Tet2−/−
k
Body temperature (°C)
Time (min)
l
Thermogenic genes in BAT
m
Thermogenic genes in iWAT
n
Thermogenic genes in eWAT

Figure S7: Leptin deficiency normalizes the metabolic changes induced by Tet2 deficiency

(a) Representative images of *Tet2*$^{+/+}$ *ob/ob* and *Tet2*$^{-/-}$ *ob/ob* mice fed HFD for 14 weeks, starting at 5 weeks of age.

(b) Representative images of fat pads and liver collected from *Tet2*$^{+/+}$ *ob/ob* and *Tet2*$^{-/-}$ *ob/ob* mice fed HFD for 14 weeks.

(c-f) Changes in oxygen consumption (c) at different time points, oxygen consumption (d) and energy expenditure (e) during light, dark hours, and full day, and daily food intake (f) after 10 weeks of HFD feeding (n = 6 *Tet2*$^{+/+}$ *ob/ob*; n = 3 *Tet2*$^{-/-}$ *ob/ob*).

(g) Changes in rectal temperatures of HFD-fed *Tet2*$^{+/+}$ and *Tet2*$^{-/-}$ mice during cold exposure (n = 5 *Tet2*$^{+/+}$; n = 4 *Tet2*$^{-/-}$).

(h-j) mRNA levels of thermogenic genes relative to *β-actin* in BAT (h), iWAT (i) and eWAT (j) of HFD-fed *Tet2*$^{+/+}$ and *Tet2*$^{-/-}$ mice (n = 4-5 mice/group).

(k) Changes in rectal temperatures of *Tet2*$^{+/+}$ *ob/ob* and *Tet2*$^{-/-}$ *ob/ob* mice during cold exposure (n = 5 *Tet2*$^{+/+}$ *ob/ob*; n = 4 *Tet2*$^{-/-}$ *ob/ob*).

(l-n) mRNA levels of thermogenic genes relative to *β-actin* in BAT (l), iWAT (m) and eWAT (n) of *Tet2*$^{+/+}$ *ob/ob* and *Tet2*$^{-/-}$ *ob/ob* mice (n = 3-5 mice/group). All data are presented as mean ± SEM. Statistical values are determined by one-way ANCOVA test in (d and e), two-sided unpaired Student's t-test in (f-n). Source data are provided as a Source Data File.

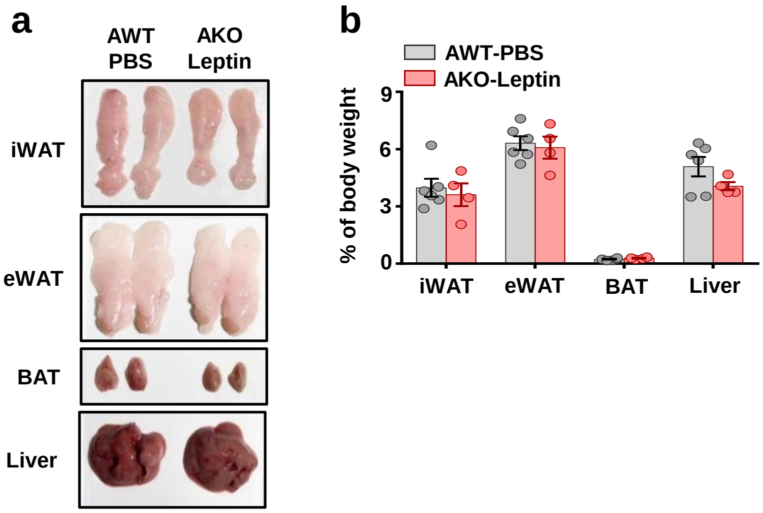

Figure S8: Adipocyte-specific Tet2 deficiency has no effect on HFD-induced fat expansion after leptin supplementation

(a) Representative images of fat pads and liver collected from HFD-fed AWT and AKO mice supplemented with PBS or leptin for 10 weeks, starting at 5 weeks of HFD.

(b) Relative tissue weights of iWAT, eWAT, BAT, and liver mass after 10 weeks of PBS or leptin supplementation (n = 6 AWT-PBS; n = 4 AKO-Leptin). All data are presented as mean ± SEM. Statistical values are determined by two-sided unpaired Student's t-test. Source data are provided as a Source Data File.

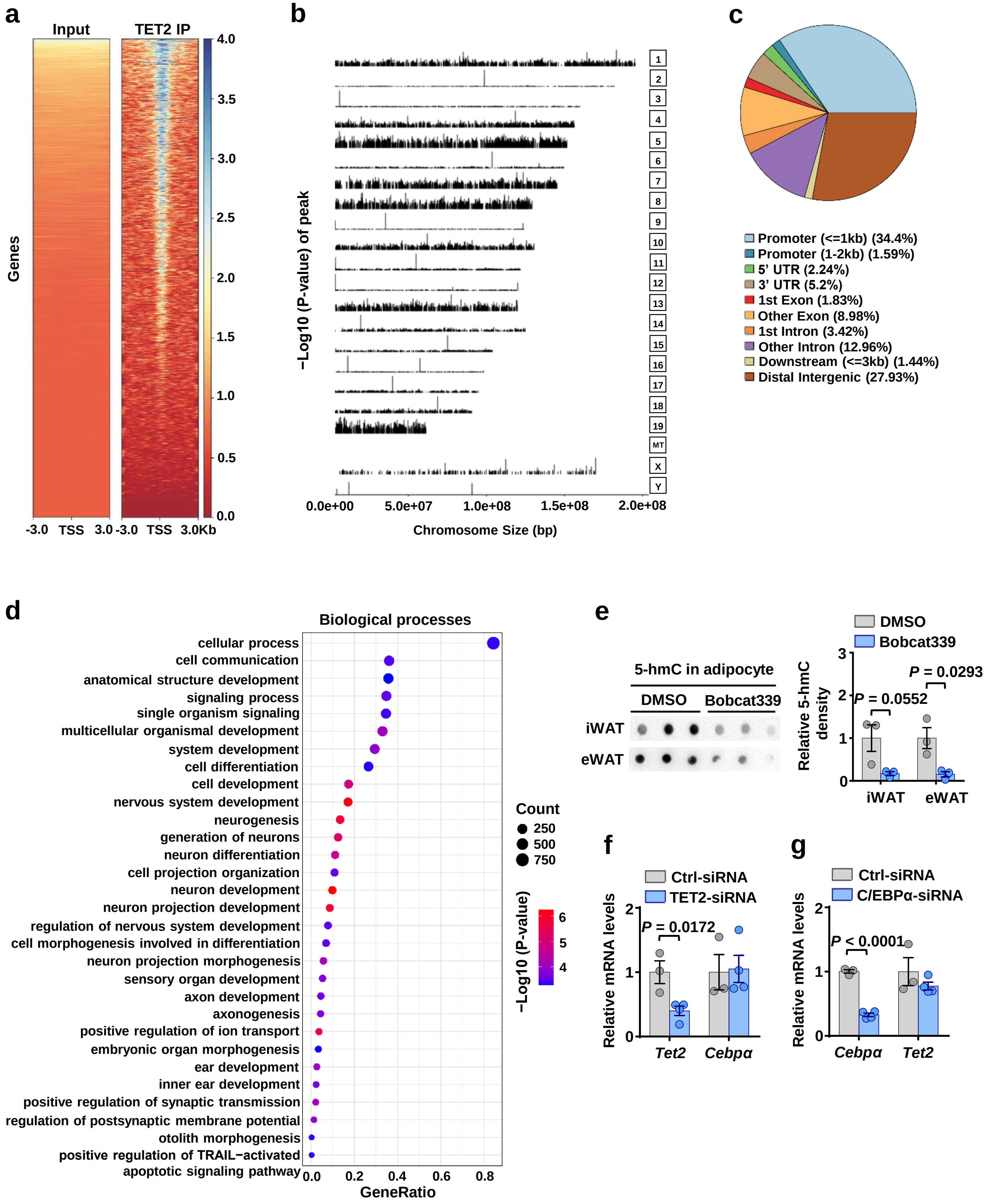

Figure S9: ChIP-seq data analysis and the interactions between C/EBPα and TET2

(a) Heatmaps of chromatin immunoprecipitation sequencing (ChIP-seq) signal for Input and TET2 antibody immunoprecipitates (TET2 IP). Red-blue color scale is used; red indicates high expression and blue indicates low expression.

(b) Ideogram of genomic location of TET2 peaks (14103 peaks) in differentiated adipocytes.

(c) Distribution of TET2 peaks over genomic elements in differentiated adipocytes.

(d) Gene ontology enrichment analysis of biological processes for the genes associated with TET2 peaks. The intensity of the color represents the *p* value, determined by a hypergeometric test.
(e) Genomic 5-hmC levels in adipocytes from iWAT and eWAT treated with DMSO or Bobcat339 for 24 h (n = 3).
(f) mRNA levels of *Tet2* and *Cebpα* relative to *36b4* in adipocytes treated with Ctrl-siRNA or TET2-siRNA for 24 h (n = 3).
(g) mRNA levels of *Tet2* and *Cebpα* relative to *36b4* in adipocytes treated with Ctrl-siRNA or C/EBPα-siRNA for 24 h (n = 3). All data are presented as mean ± SEM. *P* values are indicated on the graph. Statistical values are determined by two-sided unpaired Student's t-test. Source data are provided as a Source Data File.
