# Supplementary material for: A negative feedback loop between TET2 and leptin in adipocyte regulates body weight
Source: Nat Commun. 2024 Apr 1;15:2825. doi: 10.1038/s41467-024-46783-x (PMC10985112; doi:10.1038/s41467-024-46783-x)
Supplement: Supplementary file 3 — Description of Additional Supplementary Files [file 41467_2024_46783_MOESM3_ESM.pdf]

Description of Additional Supplementary Files:

Supplementary Data 1: TET2 ChIP-seq peak annotation data for differentiated primary adipocytes
