# Supplementary material for: A negative feedback loop between TET2 and leptin in adipocyte regulates body weight
Source: Nat Commun. 2024 Apr 1;15:2825. doi: 10.1038/s41467-024-46783-x (PMC10985112; doi:10.1038/s41467-024-46783-x)
Supplement: Supplementary file 5 — Reporting Summary [file 41467_2024_46783_MOESM5_ESM.pdf]

nature portfolio

Corresponding author(s): Tuo Deng

Last updated by author(s): Feb 17, 2024

Reporting Summary

Nature Portfolio wishes to improve the reproducibility of the work that we publish. This form provides structure for consistency and transparency in reporting. For further information on Nature Portfolio policies, see our Editorial Policies and the Editorial Policy Checklist.

Statistics

For all statistical analyses, confirm that the following items are present in the figure legend, table legend, main text, or Methods section.

n/a	Confirmed	
☐	☒	The exact sample size (*n*) for each experimental group/condition, given as a discrete number and unit of measurement
☐	☒	A statement on whether measurements were taken from distinct samples or whether the same sample was measured repeatedly
☐	☒	The statistical test(s) used AND whether they are one- or two-sided<br>*Only common tests should be described solely by name; describe more complex techniques in the Methods section.*
☐	☒	A description of all covariates tested
☐	☒	A description of any assumptions or corrections, such as tests of normality and adjustment for multiple comparisons
☐	☒	A full description of the statistical parameters including central tendency (e.g. means) or other basic estimates (e.g. regression coefficient) AND variation (e.g. standard deviation) or associated estimates of uncertainty (e.g. confidence intervals)
☐	☒	For null hypothesis testing, the test statistic (e.g. *F*, *t*, *r*) with confidence intervals, effect sizes, degrees of freedom and *P* value noted<br>*Give P values as exact values whenever suitable.*
☒	☐	For Bayesian analysis, information on the choice of priors and Markov chain Monte Carlo settings
☒	☐	For hierarchical and complex designs, identification of the appropriate level for tests and full reporting of outcomes
☐	☒	Estimates of effect sizes (e.g. Cohen's *d*, Pearson's *r*), indicating how they were calculated

Our web collection on statistics for biologists contains articles on many of the points above.

Software and code

Policy information about availability of computer code

Data collection	Applied Biosystem QuantStudio Real-Time PCR System version 1.6.1 (qPCR), OLYMPUS CX41-32RFL microscope system (immunochemistry). Western blot images were captured by Image Lab version 4.0 (Bio-Rad). The SRA files of RNA-seq were retrieved from a public database (GSE132706) utilizing the sra-tools software.
Data analysis	Image-Pro Plus 6.0 (imaging analysis). The SRA files of RNA-seq were transformed into fastq format for upstream analysis. Alignment procedures were conducted using hisat2 2.2.1 software against the mm10 version of the reference genome. Quantification of gene expression from RNA-seq data was performed using featureCounts 2.0.6 to generate a count matrix file, employing the mm10 version of the gene annotation file. The TPM (Transcripts Per Million) values were computed using R 4.2.0 software. Raw sequence quality of ChIP-seq was assessed with Fastqc software. NGS QC Toolkit was used to remove poor quality sequences, Illumina-specific sequences and adapters from the reads. Reads were aligned against reference genome GRCm39 with bowtie1. Peak calling and read density in peak regions were performed by MACS2. R package ChIPseeker was used for downstream analysis of peaks, such as annotations, visualizations binding site distribution relative to features and obtaining enriched pathways. GraphPad Prism 9 and Microsoft Office Excel 2021 (v.16.56) were used for satistical analyses.

For manuscripts utilizing custom algorithms or software that are central to the research but not yet described in published literature, software must be made available to editors and reviewers. We strongly encourage code deposition in a community repository (e.g. GitHub). See the Nature Portfolio guidelines for submitting code & software for further information.

Data

Policy information about availability of data

All manuscripts must include a data availability statement. This statement should provide the following information, where applicable:

- Accession codes, unique identifiers, or web links for publicly available datasets
- A description of any restrictions on data availability
- For clinical datasets or third party data, please ensure that the statement adheres to our policy

The bulk RNA-seq data of iWAT and eWAT used in this study are available in the GEO database under accession number GSE132706. Hyperlink: https://www.ncbi.nlm.nih.gov/geo/query/acc.cgi?acc=GSE132706. The microarray data of human adipocytes67 used in this study are available in the GEO database under accession number GSE44000. Hyperlink: https://www.ncbi.nlm.nih.gov/geo/query/acc.cgi?acc=GSE44000. ChIP-seq data of differentiated adipocytes generated in this study have been deposited in the GEO database under accession number GSE252186. Hyperlink: https://www.ncbi.nlm.nih.gov/geo/query/acc.cgi?acc=GSE252186. All other data are available in the article and its supplementary files or from the corresponding author upon request. Source data are provided with this paper.

Research involving human participants, their data, or biological material

Policy information about studies with human participants or human data. See also policy information about sex, gender (identity/presentation), and sexual orientation and race, ethnicity and racism.

Reporting on sex and gender	We have reported the sex of the subjects in all of the human studies in Supplementary Table1.
Reporting on race, ethnicity, or other socially relevant groupings	We have not reported on race, ethnicity, or other socially relevant groupings in this manuscript.
Population characteristics	Human SAT samples were collected from two groups: obese donors (BMI ≥30 kg/m2, age 30.2 ± 3.9 years) who met the recruitment criteria for bariatric surgery, and nonobese donors (BMI <30 kg/m2, , age 40.8 ± 2.7 years) undergoing non-acute cholecystectomy surgery.
Recruitment	The participants were randomly recruited at The Second Xiangya Hospital of Central South University. There is no potential self-selection bias or other biases. All participants recruited in the study signed informed consent. Obese donors (BMI ≥30 kg/m2) who met the recruitment criteria for bariatric surgery, and nonobese donors (BMI <30 kg/m2) undergoing non-acute cholecystectomy surgery.
Ethics oversight	This study was conducted in accordance with the Declaration of Helsinki and was approved by the Ethics Committee of the Second Xiangya Hospital of Central South University (No. LYF2022207). Written informed consent was obtained from all human donors prior to their enrollment in the study.

Note that full information on the approval of the study protocol must also be provided in the manuscript.

Field-specific reporting

Please select the one below that is the best fit for your research. If you are not sure, read the appropriate sections before making your selection.

☒ Life sciences ☐ Behavioural & social sciences ☐ Ecological, evolutionary & environmental sciences

For a reference copy of the document with all sections, see nature.com/documents/nr-reporting-summary-flat.pdf

Life sciences study design

All studies must disclose on these points even when the disclosure is negative.

Sample size	No statistical methods were used to predetermine sample size. Sample size was decided based on previous studies of similar experiments in the metabolism field. For animal experiments, n≥4 was chosen based on the previous publications (Meilian Liu et al, 2014, Cell Metabolism; Haiyan Zhou et al, 2021, Nature Communications). For in vitro experiments, n≥3 was chosen based on the previous publications (Tuo Deng et al.,2013, Cell metabolism) and this size is necessary to calculate statistical significances.
Data exclusions	Mice that did not respond to glucose/insulin in GTT/ITT experiments were excluded.
Replication	All experiments were, at least, twice repeated and the results were successful in replication.
Randomization	Age and gender matched mice were randomly allocated to experimental groups. For cell culture experiments, cells were randomly assigned to experimental groups. The participants were randomly recruited at The Second Xiangya Hospital of Central South University.
Blinding	Animal experiments were performed blindly by two investigators. For cell culture experiments, investigators were not blinded to treatments, because the investigators who performed the experiments was the person making the analysis. However, no subjective assessments were made. Human data were analyzed blindly because human SAT samples were collected and grouped by surgeons, who were not involved in the subsequent analysis.

Reporting for specific materials, systems and methods

We require information from authors about some types of materials, experimental systems and methods used in many studies. Here, indicate whether each material, system or method listed is relevant to your study. If you are not sure if a list item applies to your research, read the appropriate section before selecting a response.

Materials & experimental systems

n/a	Involved in the study
☐	☒ Antibodies
☐	☒ Eukaryotic cell lines
☒	☐ Palaeontology and archaeology
☐	☒ Animals and other organisms
☒	☐ Clinical data
☒	☐ Dual use research of concern
☒	☐ Plants

Methods

n/a	Involved in the study
☐	☒ ChIP-seq
☒	☐ Flow cytometry
☒	☐ MRI-based neuroimaging

Antibodies

Antibodies used

Neutralizing leptin antibody (R and D Systems, Cat# AF498, RRID:AB_355394); anti-5-mC (Active Motif, Cat#61255, RRID:AB_2783884, diluted 1:1000 for dot blot); anti-5-hmC (Active Motif, Cat#39791, RRID:AB_2630381, diluted 1:1000 for dot blot); anti-TET2 (Proteintech, Cat#21207-1-AP, RRID:AB_10734584, diluted 1:1000 for WB, diluted 1:100 for ChIP; Abcam; Cat#94580, RRID:AB_10887588, diluted 1:200 for IHC); anti-C/EBPα (GeneTex, Cat# GTX100674, RRID:AB_10618812, diluted 1:100 for ChIP; Cell Signaling Technology, Cat#8178, RRID:AB_11178517, diluted 1:1000 for WB); anti-p-STAT3 (Cell Signaling Technology, Cat#9145, RRID:AB_2491009, diluted 1:200 for IHC); anti-β-actin (Sigma-Aldrich, Cat#A5316, RRID:AB_476743, diluted 1:40000 for WB); HRP Conjugated AffiniPure Goat Anti-Rabbit IgG (Boster, Cat#BA1054, diluted 1:5000 for dot blot and WB, diluted 1:200 for IHC).

Validation

Neutralizing leptin antibody
https://www.rndsystems.com/cn/products/mouse-leptin-ob-antibody_af498
Reactivity: Mouse
Antibody Type: Polyclonal
Host Species: Goat
Product Citation: Kim, et al. 2015. Nat Immunol.

anti-5-mC
https://www.activemotif.com.cn/catalog/details/61255/5-methylcytosine-antibody-pab
Reactivity: Human, Not Species Specific
Antibody Type: Polyclonal
Host Species: Rabbit
Product Citation: Yundong H, et al. 2021. Nat Commun

anti-5-hmC
https://www.activemotif.com.cn/catalog/details/39791/5-hydroxymethylcytidine-antibody-pab
Reactivity: Human, Mouse, Not Species Specific
Antibody Type: Polyclonal
Host Species: Rabbit
Product Citation: Carolina M G, et al. 2016. Nat Commun

anti-TET2
https://www.ptgcn.com/products/TET2-Antibody-21207-1-AP.htm
Reactivity: Human, Mouse
Antibody Type: Polyclonal
Host Species: Rabbit
Product Citation: Maria A H, et al. 2019. Sci Adv
https://www.abcam.cn/products/primary-antibodies/tet2-antibody-ab94580.html
Reactivity: Human, Mouse
Antibody Type: Polyclonal
Host Species: Rabbit
Product Citation: Jiadi Lv et al. 2022. Nat Cell Biol

anti-C/EBPα
https://www.genetex.cn/Product/Detail/C-EBP-alpha-antibody-N1-N-term/GTX100674
Reactivity: Human, Mouse, Sheep
Antibody Type: Polyclonal
Host Species: Rabbit

Product Citation: Wu JS, et al. 2017. Mol Neurobiol
https://www.cellsignal.cn/products/primary-antibodies/c-ebpa-d56f10-xp-rabbit-mab/8178
Reactivity: Human, Mouse
Antibody Type: Polyclonal
Host Species: Rabbit
Product Citation: Wu SC, et al. 2022. Nat Commun

anti-p-STAT3
https://www.cellsignal.cn/products/primary-antibodies/phospho-stat3-tyr705-d3a7-xp-rabbit-mab/9145
Reactivity: Rabbit, Sheep, Mouse, Human
Antibody Type: Polyclonal
Host Species: Rabbit
Product Citation: Moro CF, et al. 2023. Nat Commun

anti-β-actin
https://www.sigmaaldrich.cn/CN/zh/product/sigma/a5316
Reactivity: Rabbit, Sheep, Mouse, Human
Antibody Type: Monoclonal
Host Species: Mouse
Product Citation: Lee EW, et al. 2012. Nat Commun

HRP Conjugated AffiniPure Goat Anti-Rabbit IgG
https://www.bosterbio.com/hrp-conjugated-goat-anti-rabbit-igg-secondary-antibody-ba1054-boster.html
Reactivity: Rabbit
Antibody Type: Polyclonal
Host Species: Goat
Product Citation: Yuxuan Yang, et al. 2023. Nat Commun

Eukaryotic cell lines

Policy information about cell lines and Sex and Gender in Research

Cell line source(s)	3T3-L1 cells were purchased from ATCC.
Authentication	3T3-L1 cells were authenticated by suppliers. This cell line was also authenticated through lipid accumulation during adipocyte differentiation and analysis of adipogenic gene expression using qPCR.
Mycoplasma contamination	3T3-L1 cells was tested negative for mycoplasma contamination.
Commonly misidentified lines (See ICLAC register)	No commonly misidentified lines were applied in this study.

Animals and other research organisms

Policy information about studies involving animals; ARRIVE guidelines recommended for reporting animal research, and Sex and Gender in Research

Laboratory animals	6-week-old C57BL/6J mice and 12-week-old C57BL/6J ob/ob (Lepob/Lepob) mice were purchased from Slac Laboratory Animal Inc and the Model Animal Research Center of Nanjing University, respectively. Heterozygous leptin knockout mouse (ob/+) mice (Stock: 19006A) were purchased from Jiangsu Wukong Biotechnology Co. LTD. Tet2−/− mice were generated and kindly provided by Dr. Xu' group. 6-week-old Adipoq-Cre mice (B6.FVB-Tg (Adipoq-Cre)1Evdr/J; Stock 028020) and Tet2fl/fl mice (B6;129S-Tet2tm1.1Iaai/J; Stock 017573) were both obtained from the Jackson Laboratory and bred to generate experimental mice groups, including Tet2fl/fl and Tet2AdipoqCre. Tet2−/− mice were crossed with ob/+ mice to generate Tet2 and leptin double knockout mice (Tet2+/+ ob/ob). Tet2+/+, Tet2−/−, AWT and AKO mice fed HFD for 12 weeks, starting at 6 weeks of age. AWT-PBS and AKO-Leptin mice supplemented with PBS or leptin for 10 weeks, starting at 5 weeks of HFD. Tet2+/+ ob/ob and Tet2−/− ob/ob mice fed HFD for 14 weeks, starting at 5 weeks of age.
Wild animals	No wild animals were used in the study.
Reporting on sex	The sex of mice used for experiments is male, as male mice exhibited a significantly higher percentage of visceral fat, body fat (Elise Jeffery, et al. Cell Metab. 2016) and weight gain (Abigail E. Salinero, et al. Int J Obes (Lond). 2018), worsened glucose tolerance and decreased insulin sensitivity (Medrikova, D., et al. Int J Obes (Lond). 2012) compared with female mice.
Field-collected samples	No field-collected samples were used in this study.
Ethics oversight	All animal studies were performed in accordance with procedures approved by the Central South University Animal Care and Use Committee.

Note that full information on the approval of the study protocol must also be provided in the manuscript.

Plants

Seed stocks	n/a
Novel plant genotypes	n/a
Authentication	n/a

ChIP-seq

Data deposition

☒ Confirm that both raw and final processed data have been deposited in a public database such as GEO.

☐ Confirm that you have deposited or provided access to graph files (e.g. BED files) for the called peaks.

Data access links<br>*May remain private before publication.*	https://www.ncbi.nlm.nih.gov/geo/query/acc.cgi?acc=GSE252186
Files in database submission	GSM7996293 ChIP<br>GSM7996294 Input<br>RAW data has been deposited in the GEO database and will be made public after publication.
Genome browser session<br>(e.g. UCSC)	*Provide a link to an anonymized genome browser session for "Initial submission" and "Revised version" documents only, to enable peer review. Write "no longer applicable" for "Final submission" documents.*

Methodology

Replicates	A cell quantity of 5×10^7 primary ASCs that were induced to differentiate into mature adipocytes, as described in the paper. Input and IP samples were generated for each condition.
Sequencing depth	Paired-end sequencing was performed to obtain 10.7 GB reads.
Antibodies	anti-TET2 (Proteintech, Cat#21207-1-AP)
Peak calling parameters	Peak calling was conducted by MACS2. The 1e-5 was the setting for the p-value option.

Data quality	Raw sequence quality was assessed with Fastqc software. NGS QC Toolkit was used to remove poor quality sequences, Illumina-specific sequences and adapters from the reads. Reads were aligned against reference genome GRCm39 with bowtie1.
Software	Aligner: bowtie1<br>Peak caller: MACS2<br>Peak annotation: R package ChIPseeker
