# Supplementary material for: A negative feedback loop between TET2 and leptin in adipocyte regulates body weight
Source: Nat Commun. 2024 Apr 1;15:2825. doi: 10.1038/s41467-024-46783-x (PMC10985112; doi:10.1038/s41467-024-46783-x)
Supplement: Supplementary file 6 — Supplementary tables [file 41467_2024_46783_MOESM6_ESM.pdf]

Table S1:

Clinical parameters	Subject population (N=15)	
	Nonobese (N=8) Mean ± SE	Obese (N=7) Mean ± SE
Age (years)	40.8 ± 2.7	30.2 ± 3.9
Sex (Female/Male)	4/4	3/4
BMI (kg/m^2)	23.1 ± 1.3	37.2 ± 3.8$^{*p = 0.0002}$
TC (mmol/L)	3.7 ± 0.3	3.9 ± 0.2
TG (mmol/L)	1.0 ± 0.2	2.0± 0.5$^{*p = 0.0277}$
HDL (mmol/L)	1.1 ± 0.1	0.9 ± 0.1
LDL (mmol/L)	2.2 ± 0.2	2.6 ± 0.2$^{*p = 0.0313}$

Table S1. Phenotypic parameters of the nonobese (BMI <30 kg/m^2) and obese (BMI ≥30 kg/m^2) human subjects. Asterisks indicate significant differences ($P < 0.05$ by two-sided unpaired Student's t-test) between nonobese and obese subjects. Phenotype abbreviations: body mass index (BMI), total cholesterol (TC), triglycerides (TG), high-density lipoprotein cholesterol (HDL) and low-density lipoprotein cholesterol (LDL). Data represent Means±SEM.

Table S2: siRNA primer sequences

Genes	Primers (5'-3')
Jak2	GGAGAGTATCTGAAGTTTC
Stat3	CCACGTTGGTGTTTCATAA
C/EBPα	AGAAGTCGGTGGACAAGAA
SP1	GGATGGTTCTGGTCAAATA
Tet2	GTCACTGCATGTTTGGACTTCTCTG

Table S3: mRNA primer sequences (m: mouse; h: human)

Genes	Primers (5'-3')
m36b4	Forward: TTATAACCCTGAAGTGCTCGAC
	Reverse: GTAAGTGGGAAGGTGTACTCAG
mβ-actin	Forward: ACCGTGAAAAGATGACCCAG
	Reverse: GAGCATAGCCCTCGTAGATG
mTet1	Forward: ACATCCCACAGACCGAAGAT
	Reverse: TTCTGGGGTTTTCACTCCTC
mTet2	Forward: CTCGCATCAGAACTCTACTCAG
	Reverse: GTCTCTGGATTCTCACCCTG
mTet3	Forward: GAACTCATGGAGGATCGGTATGGA
	Reverse: CAGCTTCTCCTCCAGTGTGTGTCTT
mStat3	Forward: TGGAAGAGGCGGCAGCAGATAGC

Genes	Primers (5’-3')
	Reverse: GCACGGCCCCCATTCCCACAT
mJak2	Forward: CGGCATGATTTTGTTCACGG
	Reverse: GATAGTCTTGGATCTTCGCTCG
mDio2	Forward: CAGTGTGGTGCACGTCTCCAATC
	Reverse: TGAACCAAAGTTGACCACCAG
mUcp-1	Forward: AGAAGGATTGCCGAAACTGTAC
	Reverse: GCCACACCTCCAGTCATTAAG
mCidea	Forward: TGACATTCATGGGATTGCAGAC
	Reverse: GGCCAGTTGTGATGACTAAGAC
mPrdm16	Forward: ACAGGCAGGCTAAGAACCAG
	Reverse: CGTGGAGAGGAGTGTCTTCAG
mPpargc1a	Forward: ACTGAGCTACCCTTGGGATG
	Reverse: TAAGAATTTCGGTGGTGACA
mAdiponectin	Forward: TGTTCCTCTTAATCCTGCCCA
	Reverse: CCAACCTGCACAAGTTCCCTT
mPPARg	Forward: TCGCTGATGCACTGCCTATG
	Reverse: GAGAGGTCCACAGAGCTGATT
mCebpa	Forward: CAAGAACAGCAACGAGTACCG
	Reverse: GTCACTGGTCAACTCCAGCAC
mFabp4	Forward: AAGGTGAAGAGCATCATAACCCT
	Reverse: TCACGCCTTTCATAACACATTCC
mLeptin	Forward: GTGCCTATCCAGAAAGTCCAG
	Reverse: GACCTGTTGATAGACTGCCAG
hβ-ACTIN	Forward: ACCACACCTTCTACAATGAGC
	Reverse: GCGTACAGGGATAGCACAG
hTET2	Forward: CTTTCCTCCCTGGAGAACAGCTC
	Reverse: TGCTGGGACTGCTGCATGACT
hLEPTIN	Forward: CAAGATGACACCAAAACCCTC
	Reverse: ACGTTTCTGGAAGGCATACTG
